# Mosquito Host Seeking in 3D Using a Versatile Climate-Controlled Wind Tunnel System

**DOI:** 10.3389/fnbeh.2021.643693

**Published:** 2021-03-11

**Authors:** Annika Hinze, Jörgen Lantz, Sharon R. Hill, Rickard Ignell

**Affiliations:** ^1^Disease Vector Group, Chemical Ecology, Department of Plant Protection Biology, Swedish University of Agricultural Sciences, Alnarp, Sweden; ^2^Jörgen Lantz Engineering Consulting Firm, Alnarp, Sweden; ^3^Max Planck Centre Next Generation Chemical Ecology, Uppsala, Sweden

**Keywords:** *Anopheles gambiae*, host seeking, 3D tracking, carbon dioxide, olfaction, human odor, behavior

## Abstract

Future anthropogenic climate change is predicted to impact sensory-driven behaviors. Building on recent improvements in computational power and tracking technology, we have developed a versatile climate-controlled wind tunnel system, in which to study the effect of climate parameters, including temperature, precipitation, and elevated greenhouse gas levels, on odor-mediated behaviors in insects. To establish a baseline for future studies, we here analyzed the host-seeking behavior of the major malaria vector mosquito, *Anopheles gambiae sensu strico*, to human odor and carbon dioxide (CO_2_), under tightly controlled climatic conditions, and isolated from potential background contamination by the presence of an experimenter. When presented with a combination of human foot odor and CO_2_ (case study I), mosquitoes engaged in faster crosswind flight, spent more time in the filamentous odor plume and targeted the odor source more successfully. In contrast, female *An. gambiae s. s*. presented with different concentrations of CO_2_ alone, did not display host-seeking behavior (case study II). These observations support previous findings on the role of human host-associated cues in host seeking and confirm the role of CO_2_ as a synergist, but not a host-seeking cue on its own. Future studies are aimed at investigating the effect of climate change on odor-mediated behavior in mosquitoes and other insects. Moreover, the system will be used to investigate detection and processing of olfactory information in various behavioral contexts, by providing a fine-scale analysis of flight behavior.

## Introduction

Insects integrate cues of multiple sensory modalities to navigate in their environment in order to locate suitable food sources, mating partners, or oviposition sites (Buehlmann et al., 2020). Understanding insect flight behavior in response to their variable olfactory environment requires an experimental system that is able to mimic the required climatic conditions in a precise manner, while facilitating easy presentation of cues, observation, and analysis of flight behavior in detail. The versatile climate chamber and wind tunnel system presented in this study provides these features, and also facilitates tracking insect flight in 3 dimensions (3D).

Female mosquitoes rely predominantly on odors to find a blood meal, especially at longer distances from the host, while also using visual and thermal cues when nearing the target (Takken and Knols, 1999; Cardé, 2015; Raji and DeGennaro, 2017). Our understanding of host-seeking behavior in mosquitoes has expanded substantially due to advancements in video capture, tracking technology and computational power (Anderson and Perona, 2014; Spitzen and Takken, 2018; Manoukis and Collier, 2019). In the recent past, tracking mosquito behavior has allowed for analyses in greater detail, and provided new levels of understanding in host-seeking strategies, the different sensory cues involved and their integration (Dekker and Cardé, 2011; Lacey and Cardé, 2011; Lacey et al., 2014; McMeniman et al., 2014; van Breugel et al., 2015; Hawkes and Gibson, 2016). Moreover, behavioral responses to mosquito vector control tools that are targeting host-seeking behavior, such as insecticide-treated bed nets and baited traps, have been analyzed to improve their efficiency (Cooperband and Cardé, 2006; Spitzen et al., 2014; Parker et al., 2015, 2017; Angarita-Jaimes et al., 2016; Cribellier et al., 2018, 2020; Amos et al., 2020).

Based on tracking studies and behavioral observations, the long-range flight strategy of female anthropophilic mosquitoes, such as the African malaria vector, *Anopheles gambiae sensu lato*, and the yellow fever mosquito, *Aedes aegypti*, in response to human host odors has been characterized as “cast and surge,” in which mosquitoes surge upwind upon contact with an odor-laden filament of air and perform crosswind flight if the trace is lost (Cardé and Willis, 2008; Dekker and Cardé, 2011; Spitzen et al., 2013). At intermediate distances, gated by the encounter of human-emanated carbon dioxide (CO_2_) and body odors, mosquitoes approach high-contrast visual features (van Breugel et al., 2015; Hawkes and Gibson, 2016) and initiate landing in the presence of short-range host cues, such as body heat and humidity (McMeniman et al., 2014). While these basic characteristics are common to all host-seeking mosquito species, details, such as the relative importance of the respective cues, differ in respect to, e.g., host preference and daily flight activity patterns (Cooperband and Cardé, 2006; Dekker and Cardé, 2011; Spitzen et al., 2013; Hawkes and Gibson, 2016). In *An. gambiae*, for instance, the role of CO_2_ in regulating host seeking is controversial. While some studies found CO_2_ on its own to be a host-seeking cue, eliciting activation, orientation, or both (Healy and Copland, 1995; Lorenz et al., 2013), others did not find such an effect (de Jong and Knols, 1995; Takken et al., 1997; Spitzen et al., 2008). This discrepancy can partially be explained by differences in behavioral assays used and the mode of presentation of CO_2_, but also contamination by odors from an experimenter cannot be excluded in some studies (Webster et al., 2015).

The improved wind tunnel system presented in this study is equipped with a highly-versatile automated climate-control that allows us to analyse the odor-mediated anemotaxis of *An. gambiae sensu stricto* in response to human host odors under stable and precise climatic conditions, while reducing background odors to a minimum. The case studies presented here investigate the role of human host cues in *An. gambiae s. s*. host seeking. Case study I confirms that, when presented with a salient odor, i.e., a combination of human odor and CO_2_, mosquitoes spend more time in the filamentous odor plume, engage in faster crosswind flight maneuvers and find the source more reliably. Case study II supports previous findings that *An. gambiae s. s*. likely does not use CO_2_ on its own as a cue in host seeking.

## Materials and Methods

### Wind-Tunnel System

#### Hardware: Air Treatment, Climate Chamber, Wind Tunnel

**Figure 1:**
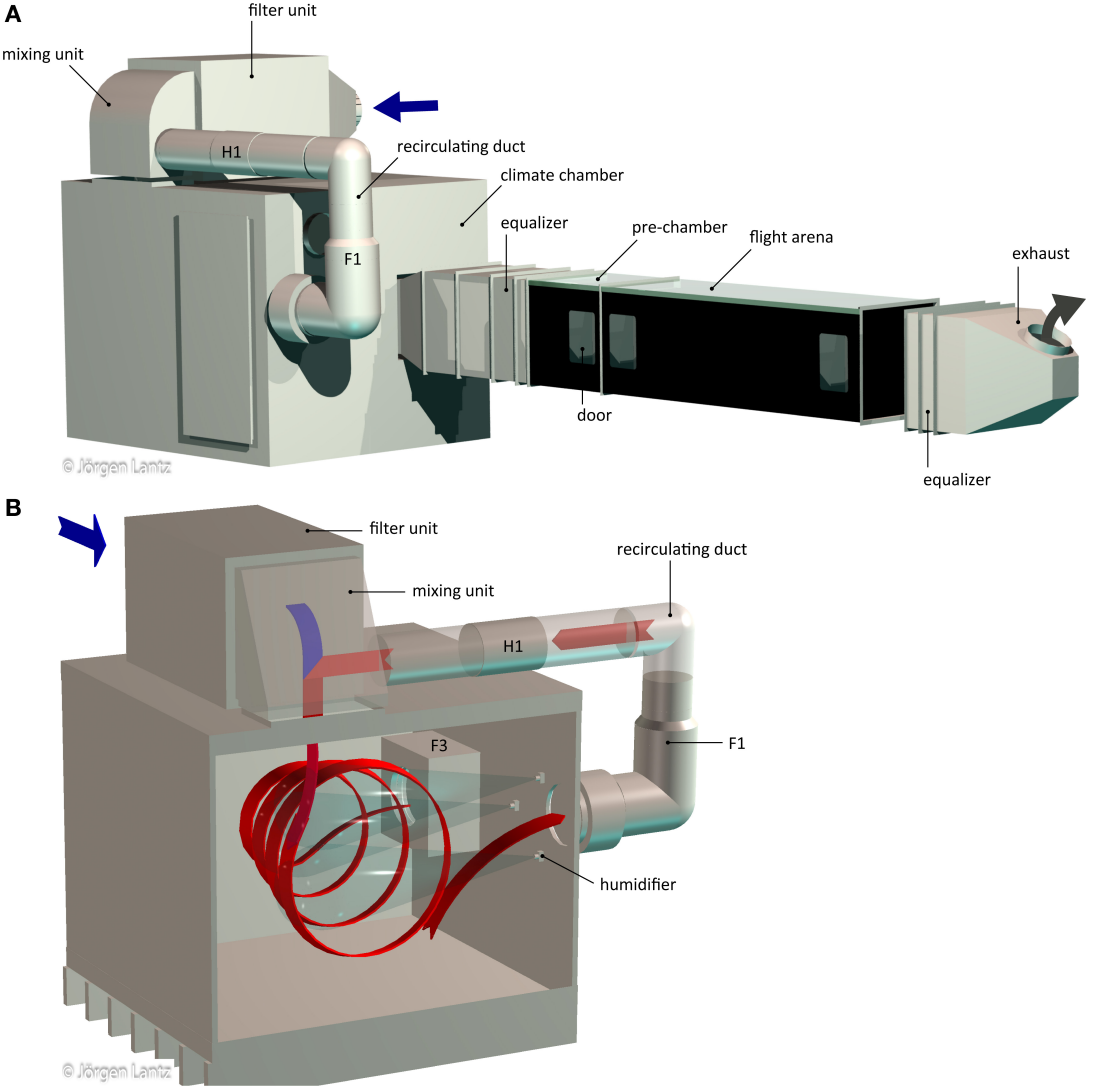
Schematic of the wind tunnel system **(A)** and climate chamber **(B)**. Incoming air (blue) is filtered in the filter unit and passed on to the mixing unit where it is mixed with warm air (red) from the recirculating duct. In the climate chamber, the air is humidified by five humidifiers (three shown) and passed on through an equalizer toward the pre-chamber and flight arena. Both pre-chamber and flight arena can be accessed by doors. Air is removed by the exhaust. F1 and F3 indicate the fans and H1 the heater. F2 and the flow control valve are placed on the feeding line to the wind tunnel system and thus not shown.

Air for the wind-tunnel system was supplied from the ventilation system of the building, pre-filtered, with a low, constant pressure and a temperature of 20–22°C. Airflow within the wind tunnel system was regulated by two circular duct fans (F2: K 315 sileo, F3: KV 315 sileo; Systemair, Skinnskatteberg, Sweden) and a mechanical flow control valve (BDEP-4-025-1; FläktGroup, Herne, Germany), equipped with a modulating damper actuator (LM24A-SR; Belimo, Hinwil, Switzerland) that is adjusted from the control panel of the wind tunnel (Figure 1; F2 and the flow control valve are installed on the feeding line before the filter unit and thus not shown). Incoming air to the wind-tunnel system was pushed through a Camfil filter unit equipped with a pre-filter and 16 activated carbon filters (pre-filter: EcoPleat Eco 3GPF ePM1 55% 592x592x48-F7 ISO; carbon filters: CamCarb CM 2600 GC VOC; ducted filter housing: CamCube HF-CC 1010 1010AZ; Camfil, Stockholm, Sweden) before entering the mixing unit. In the mixing unit, turbulent intermixing of the fresh air from the filter unit and recirculating air from the climate chamber occurs and mixed air is passed into the climate chamber (Figure 1).

Within the climate chamber (stainless steel, l × w × h: 1,760 mm × 2,000 mm × 1,570 mm; Figure 1), air temperature and relative humidity (RH) can be adjusted up to 27.0°C and 70 % RH, respectively, regulated from the control panel. The lower limits of both parameters are determined by the air fed into the wind-tunnel system from the ventilation system of the building. Within the climate chamber, the air is humidified by five humidifiers (B 1/4 ML-1.5; Spraying Systems Co, Wheaton, IL, US) that are placed in the zone of recirculating air in the chamber and fed by the warm water supply of the building (Figure 1B). Connections for cold water, distilled water and pressurized air are installed and can be used for future applications, e.g., to adjust temperature and humidity to values different from those specified above. The climate chamber with its large inner dimensions is constructed such that it both facilitates turbulent intermixing of the air and permits easy servicing, as well as placing of additional equipment for e.g., raising ozone and CO_2_ background levels. The climate chamber is built in a stainless steel tray (fold height 20 mm) and placed on a waterproofing membrane (Biltema, Helsingborg, Sweden) to protect the floor from humidity. Climate chamber walls are thermally insulated with styrofoam (thickness 40 mm) and covered with a waterproofing membrane. The recirculating duct, which is constantly passing a part of the warm, humidified air from the climate chamber back to the mixing unit (Figure 1B), is equipped with an in-line duct fan (F1: KV 315 sileo; Systemair) and a circular electric duct heater (H1: CV25-60-M; VEAB Heat Tech AB, Hässleholm, Sweden), regulated by the control panel. The climate chamber and the majority of other parts are made from stainless steel, except fans, filter house and heater.

**Figure 2:**
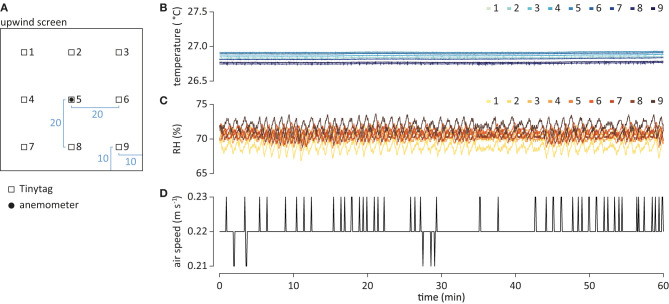
Physical parameters of the wind tunnel system. **(A)** Schematic of the placement of the Tinytags (open square), for measurement of temperature and relative humidity (RH), and anemometer (filled circle), for wind speed, at the upwind screen. Measurements in cm. The distance to the upwind screen was 15 cm. Temperature **(B)**, RH **(C)**, and air speed **(D)** over time.

From the climate chamber to the pre-chamber and flight arena, the air passes through an equalizer, in which variations in temperature, humidity, and speed of the airflow are stabilized (6 perforated metal sheet units; 1,000 mm × 600 mm × 600 mm; Figure 1A). The near-laminar airflow entering the pre-chamber and flight arena is stable in temperature, RH, and speed (methods see below; Figure 2). Within the pre-chamber (600 mm × 600 mm × 600 mm), the air passing toward the adjacent flight arena (2,000 mm × 600 mm × 600 mm) can be manipulated, e.g., by introducing an odor source as demonstrated in this study. The bodies of both chambers are made from black polycarbonate (thickness 5 mm), each covered with a transparent, removable acrylic glass roof (thickness 6 mm). The matt surface of the black polycarbonate body of the flight arena limits light reflections. Three doors enable access to the setup with minimal disturbance to the airflow (Figure 1A). The flight arena is closed off on both sides by black mosquito netting (mesh size 1.4 × 1.6 mm, plastic-covered fiberglass; Biltema), set in a black metal frame. Downwind of the wind tunnel, the air exits through an exhaust equipped with an equalizer unit (3 perforated metal sheet units; 400 mm × 610 mm × 610 mm, placed 280 mm away from the wind tunnel. The airspeed of the exhaust is adjusted to ca. 0.5 m s^−1^, which removes air from both the wind tunnel and the room in which it is placed.

#### Control Panel: Airspeed, Temperature and Relative Humidity

The coarse setting for the air pressure was pre-set upon installation of the wind tunnel system, where the fans F1, F2, and F3 were individually adjusted via three five-step transformers (Systemair 5000, type RE 1.5, Tuvfassons 7886-009; Tuvfassons, Sigtuna, Sweden; Figure 1). Upon operation of the wind tunnel system, wind speed can be finely regulated by an airflow damper, which is operated from the control panel. With the fixed pre-set adjustment of F1, F2, and F3, the airspeed can then be adjusted to up to 0.35 m s^−1^.

Temperature and RH are controlled via the control panel, mainly by a custom-programmed PLC unit (Millenium 3 Essential CD20- 12I/8O S 24VDC; Crouzet, Valence, France; Jörgen Lantz Engineering Consulting Firm; Supplementary Figure 1). In short, the control panel is integrating set points, actual values and input of e.g., time of ventilation and drain flushing, limits for set points and actual values, conditions for starting the wind tunnel (e.g., air flow from the building), and the control of the flow adjustment damper.

Upon shutting down the wind tunnel, an ejector drain flush is automatically activated by the control panel that flushes remaining water from the climate chamber using pressurized air. Then, the wind tunnel is dehumidified by running at maximum speed (0.35 m s^−1^) for 12 h. This removes the remaining water from the climate chamber and humidity from associated parts of the setup.

#### Quantification of Physical Parameters Within the Flight Arena

Air temperature and RH were quantified using Tinytag Plus 2 TGP-4500 data loggers (Intab, Stenkullen, Sweden), set to 1 Hz sampling rate. Tinytags were arranged in an array (Figure 2A), and placed 15 cm downwind of the upwind screen. Air speed was measured using a ThermoAir3 hot wire anemometer (Schiltknecht Messtechnik AG, Gossau, Switzerland), read every 5 s. The anemometer was placed mid-center in the flight arena, 15 cm downwind of the upwind screen (Figure 2A). Air temperature, RH and speed were recorded for an hour (Figures 2B–D).

### Case Studies

#### Mosquitoes

*Anopheles gambiae sensu stricto* (G3 strain) were reared as previously described (Omondi et al., 2015). Adult mosquitoes were maintained in Bugdorm cages (30 × 30 × 30 cm; MegaView Science, Taichung City, Taiwan) at 27 ± 1°C and 65 ± 5% RH under a 12 h light: 12 h dark regimen, and provided with 10 % sucrose *ad libitum*. For colony maintenance, adult females were fed on donor sheep blood (Håtunalab, Bro, Sweden) using a membrane feeding system (Hemotek Ltd, Blackburn, UK). For oviposition, wet filter papers were provided, and eggs transferred to larval trays (24 × 18 × 7.5 cm, filled with 2 cm of distilled water) before hatching. Larvae were fed daily on Tetramin Baby fish food (Tetra GmbH, Melle, Germany). For experiments, pupae were collected and transferred to Bugdorm cages (17.5 × 17.5 × 17.5 cm) prior to eclosion. Experiments were conducted with non-blood-fed females at 4 days post-eclosion (4 dpe). Prior to the experiment, females were sugar starved either for 4–16 h without (case study II), or 15–23 h with *ad libitum* access to water (case study I), and then transferred to individual release cages (10 × 7 cm), at least 30 min before the start of the experiment, using a mouth aspirator. Host-seeking females were pre-selected by placing a gloved hand on the netting of the cage. All experiments were conducted within the peak activity period of host seeking, i.e., the first 4 h of the scotophase (e.g., Jones and Gubbins, 1978).

#### Flight Arena

**Figure 3:**
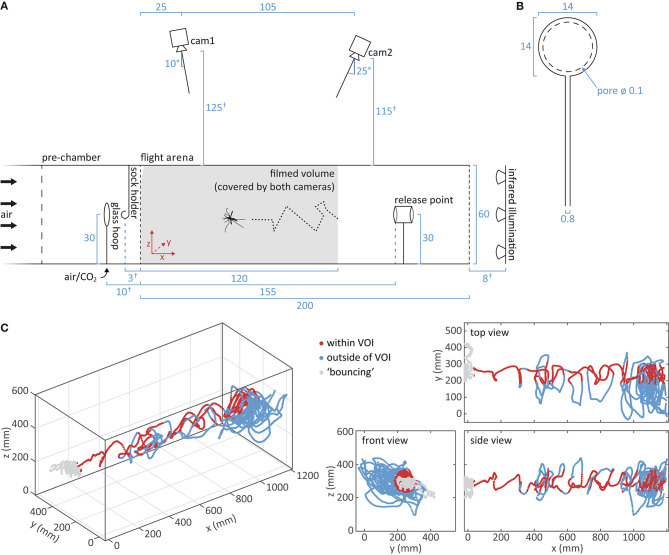
Tracking of mosquito flight. **(A)** Schematic of the flight arena and the setup for video capture of mosquito flight. A mosquito, released at the release point, flies upwind toward the upwind screen. Cameras mounted above the flight arena record the reflection of the infrared light on the body of the mosquito. The filmed volume, i.e., the area covered by both cameras, is shown in gray. Distance measurements are given in cm. Dimensions drawn to scale, apart from measurements marked by †. **(B)** Schematic of the glass hoop used for turbulent presentation of CO_2_ (Dekker and Cardé, 2011). Measurements are given in cm. **(C)** A representative flight trajectory of a single mosquito in response to carbon dioxide and human foot odor, viewed from different angles. Positions within the cylindrical volume of interest (VOI) are indicated in dark red, whereas positions outside the VOI are in light blue. “Bouncing” at the upwind screen is denoted in gray.

Mosquito flight behavior was tracked in the wind tunnel setup described above (Figures 1A, 3A). The wind tunnel was adjusted to 27.0°C and 70% RH, and the wind speed was set to 0.22 m s^−1^.

#### Odor Stimuli

Odor stimuli were delivered from two different devices, a glass hoop and a metal sock holder, placed within the pre-chamber of the wind-tunnel system (Figures 3A,B). Carbon dioxide of either ambient or elevated concentrations (1,200, 2,400, 4,800 ppm) were presented using a glass hoop with equidistant holes to create a turbulent plume (Dekker et al., 2001), which was positioned in the pre-chamber, 10 cm upwind of the upwind screen. For elevated CO_2_ concentrations, pure, pressurized CO_2_ (Strandmöllen AB, Ljungby, Sweden) was mixed in different proportions with carbon-filtered and humidified ambient air at a resulting flow rate of 1 l min^−1^. The concentration of the resulting mix was measured using a LI-820 CO_2_ analyser (LICOR Biosciences, Lincoln, NE, US) and adjusted to the desired concentration ± 50 ppm prior to entering the glass hoop. Compliance to a tolerance interval of ± 200 ppm was confirmed after each trial, as the pressure of the pure CO_2_ showed minor shifts over time. Addition of CO_2_ did not detectibly increase the flow rate of the air passing toward the glass hoop (BA-4AR flow meter; Kytola Instruments, Muurame, Finland). For the presentation of human foot odor, socks worn by the experimenter were used, in accordance with previous studies (e.g., Njiru et al., 2006; Verhulst et al., 2011; Robinson et al., 2018). Black cotton socks were worn for 19–21 h prior to the experiment, rolled up and then suspended from a metal hook between the glass hoop and the upwind screen (Figure 3A). Used socks provided an odor source for a maximum of 1 h, and were later washed with a low-perfumed washing detergent (ICA Skona, Solna, Sweden) before reuse. Here, cotton socks were chosen over nylon socks, as the foot odor collected on the former elicited responsiveness from a higher proportion of mosquitoes (enter the filmed volume; data not shown).

Odor stimuli presented in case study I were combinations of either air or 1,200 ppm CO_2_, and human foot odor or corresponding controls, i.e., no sock and clean sock. The resulting treatment combinations were “air/air,” “CO_2_/air,” “air/clean sock,” “air/used sock,” and “CO_2_/used sock.” In case study II, CO_2_ on its own was presented at either ambient (400 ppm to 445 ppm), or elevated concentrations (1,200, 2,400, 4,800 ppm). The order of treatments was randomized over the experimental day.

#### Experimental Procedure

For each trial, a release cage containing a single female mosquito was placed at the release point close to the upwind end of the wind tunnel (Figure 3A). Mosquitoes were exposed to the odor stimulus during acclimatization to avoid disturbing the air current and the mosquitoes once the trial started. After an acclimatization period of 2 min, the video recording was started, and the door of the release cage gently opened. Individuals that did not enter the filmed volume of the flight arena (Figure 3A, gray area) within 3 min were removed from further analysis. Flight behavior of responding mosquitoes was recorded until landing on the upwind screen for at least 5 s, or for up to 10 min of continuous flight. After each trial, the response (“flight,” “no flight”) and landing site (“upwind screen,” “other,” “not landing”) was visually observed. Each mosquito was only tested once. Surgical gloves were worn during the experiment, and equipment and mosquitoes were handled with great care to avoid contamination with human odor.

#### Video Capture and Flight Trajectory Reconstruction

**Figure 4:**
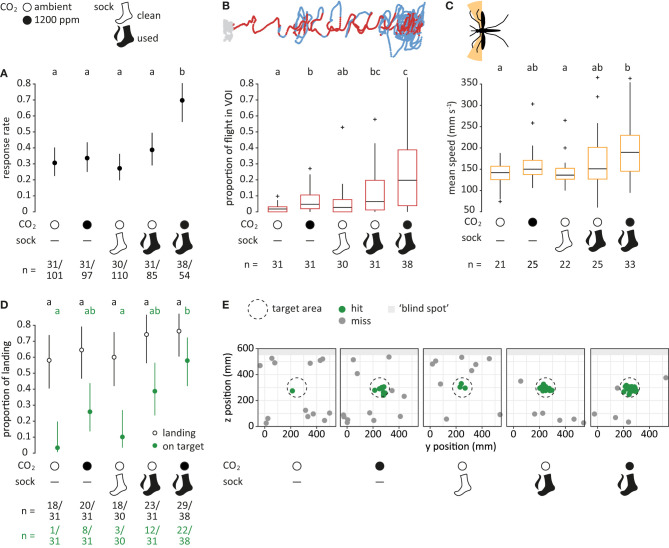
Mosquito flight in response to human host cues. **(A)** Proportion of mosquitoes responding (dots), i.e., entering the filmed volume within 3 min, for each treatment. Bars denote 95% confidence intervals. Different letters denote significant differences between the treatments (pairwise comparison using “emmeans,” corrected with the Tukey method). Sample sizes are given below the plot. **(B)** Proportion of flight in volume of interest (VOI), as shown in dark red in the top panel (see Figure 3C). Boxes represent upper and lower quartiles, whiskers denote 1.5 times interquartile distance, crosses outliers, and black horizontal lines the median. Different letters denote significant differences between the treatments (Dunn Kruskal-Wallis pairwise comparison test, Benjamini-Hochberg corrected). **(C)** Mean speed of crosswind flight for heading angles between 90° to 120° and 240° to 270° (orange area in the top panel). Only mosquitoes that were within the VOI at least once were taken into account. **(D)** Proportion of mosquitoes landing (black, open) and of mosquitoes landing on the target (green, filled) relative to the total number of responsive mosquitoes. **(E)** Landing location on the upwind screen. The defined target area (ø 15 cm) is denoted as a dashed circle. Green dots indicate landing positions within, and gray dots positions outside of the target area. Light gray area denotes the “blind spot” where tracking of landing was not possible.

**Figure 5:**
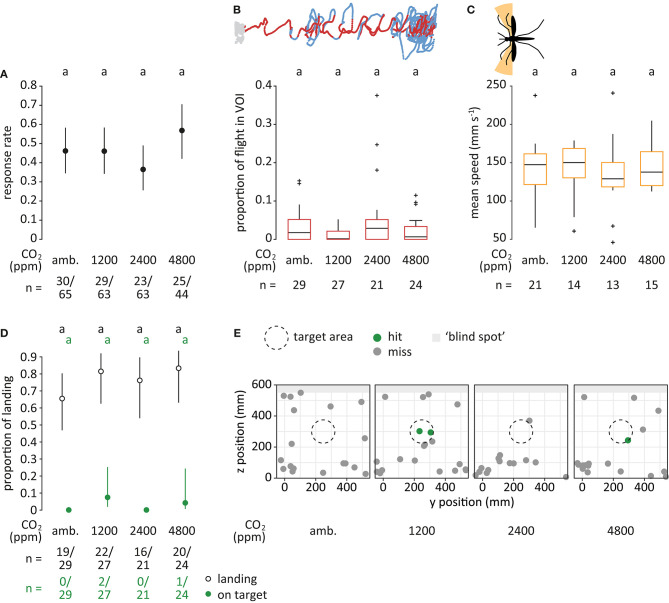
Mosquito flight in response to different concentrations of carbon dioxide. **(A)** Proportion of mosquitoes responding (dots), i.e., entering the filmed volume within 3 min, for each treatment. “amb.” for ambient. Bars denote 95% confidence intervals. Different letters denote significant differences between the treatments (pairwise comparison using ‘emmeans,' corrected with the Tukey method). Sample sizes are given below the plot. **(B)** Proportion of flight in volume of interest (VOI), as shown in dark red in the top panel (see Figure 3C). Boxes represent upper and lower quartiles, whiskers denote 1.5 times interquartile distance, crosses outliers, and black horizontal lines the median. Different letters denote significant differences between the treatments (Dunn Kruskal-Wallis pairwise comparison test, Benjamini-Hochberg corrected). **(C)** Mean speed of crosswind flight for heading angles between 90° to 120° and 240° to 270° (orange area in the top panel). Only mosquitoes that were within the VOI at least once were taken into account. **(D)** Proportion of mosquitoes landing (black, open) and of mosquitoes landing on target (green, filled) relative to the total number of responsive mosquitoes. In treatments where no mosquitoes landed on target, CIs were infinite and thus not shown. **(E)** Landing location on the upwind screen. The defined target area (ø 15 cm) is denoted as a dashed circle. Green dots indicate landing positions within, and gray dots positions outside of the target area. Light gray area denotes the “blind spot” where tracking of landing was not possible.

Flight behavior was recorded from above the wind tunnel with two infrared light (IR) sensitive GigE cameras (acA1300-60gm; Basler AG, Puchheim, Germany; Figure 3A), equipped with 4.4–11 mm lenses (LMVZ4411; Kowa, Aichi, Japan), at 60 frames s^−1^ using Media Recorder 4.0 (Noldus Information Technology, Wageningen, The Netherlands). Illumination was provided by six IR arrays (850 nm; VAR2-i2-1 IR illuminators; VAR-i2-LENS-6025 diffuser lenses; Raytec, Ashington, UK) placed at the downwind end of the flight arena (Figure 3A). Cameras recorded the reflection of the IR light on the wings and body of the mosquito. An LED array, shielded with a paper screen, at the upwind end of the wind tunnel, provided diffuse visible white light of low intensity (<1 lux; LX-101 lux meter; Lutron Electronic Enterprises, Taiwan) for visual orientation of the mosquito. Cameras were mounted at an angle above the wind tunnel, resulting in a coverage of the entire volume of the upwind 120 cm of the wind tunnel. A narrow volume at the top of the upwind screen (triangular intersection, 2.2 × 11.5 cm) was shielded by the frame holding the netting, where mosquitoes could only be observed by one camera and therefore not be tracked in 3D (Figure 3A). Due to the mosquito's protruding abdomen and hind legs while sitting, landing could be tracked except the top 5 cm of the upwind screen (Figures 4E, 5E).

EthoVision XT 14 (Noldus Information Technology) was used to convert the video files from both cameras to 2D position data. For all trials, the data was manually inspected during the process to exclude frames with identification errors. Data was generated without interpolation of missing samples or smoothing of the flight path. The 2D position data was then combined into a 3D flight path using Track3D (Noldus Information Technology; see Spitzen et al., 2013). The system was calibrated using a customized calibration frame and CentroidFinder software (Noldus Information Technology) at the start of the experimental series and if required, i.e., when the daily mean intersection error exceeded a threshold of 2.0 pixels. The following variables were calculated by Track3D and used in subsequent analysis: position in three dimensions (x, y, z), flight speed and heading angle in the vertical plane.

#### Analysis of Response Rate and Flight Trajectories

A mosquito was considered responsive if it entered the filmed volume within 3 min. Treatment factor effects were tested using a binomial generalized linear model (GLM), followed by a Chi-square test (R, version 3.5.1; R Core Team, 2018). *Post-hoc* pairwise comparisons of the treatment combinations were tested with the “emmeans” package (R), corrected using the Tukey method.

Obtained 3D trajectory data was processed and analyzed using customized Matlab (version R2020a; MathWorks, Natick, MA, US) and R scripts (version 3.5.1). In a first step, the analysis window of individual trajectories was defined and frames containing outliers were excluded. The start of the analysis window was determined by the mosquito entering the filmed volume, and the end by either the instance of landing or a maximum flight duration of 10 min. Landing was identified by detecting the time point at which the mean speed over 60 frames was below a threshold of 50 mm s^−1^ for three consecutive seconds, which was also confirmed by visual observation. Landing coordinates were determined for future analysis. In very few cases, the video recording was ended before the above criteria were fulfilled, and in these cases those files were excluded from further analysis. Data points where the mosquito's position was <6 cm away from the upwind screen were excluded from most further analyses since the physical boundary likely affected mosquito flight (“bouncing”).

For analyzing mosquito flight in the volume where it may encounter odor filaments, a volume of interest (VOI) was defined, and approximated to be a cylinder in space, with a diameter of 14 cm, centered within the flight arena (Figure 3C), based on the shape and dimension visualized by smoke paper (Günther Schaidt SAFEX Chemie GmbH, Tangstedt, Germany; Supplementary Figure 2). The proportion of flight in the VOI was calculated by the number of frames with a position within the VOI divided by the total number of frames. A Dunn Kruskal-Wallis multiple comparison *post-hoc* test with Benjamini-Hochberg correction was used for pairwise comparison between the treatments (“FSA” package; R, version 3.5.1).

Crosswind flight was quantified using the mosquito's heading angle, which is defined as the angle between the x-axis (direction of air movement) and the direction of mosquito flight in the vertical plane, in which 180° corresponds to straight upwind flight. The mean speed of crosswind flight per mosquito was calculated for heading angles between 90° to 120° and 240° to 270°. Only flight trajectories that were within the VOI at least once were considered for analysis. For pairwise comparison between the treatments, a Benjamini-Hochberg corrected Dunn Kruskal-Wallis multiple comparison *post-hoc* test was used.

Mosquito-landing response was analyzed by determining whether the landing coordinates were within a target area on the upwind screen. The target area was circular, 15 cm in diameter and centered downwind of the odor delivery devices. Treatment factor effect was tested using a binomial GLM and Chi-square test. For multiple pairwise comparisons between the treatments, the “emmeans” package was used (corrected using the Tukey method).

## Results

### Case Study I—Human Host Cues

#### Response Rate

Of the 447 mosquitoes tested, 161 responded by entering the filmed volume within 3 min after opening the door of the release cages. Human host cues had a significant effect on the number of mosquitoes responding, in which both factors, CO_2_ and human foot odor, and their interaction, contributed significantly to the observed effect (Chi-square test, *p* < 0.05). A significantly larger proportion of mosquitoes (ca. 70%) entered the filmed volume when exposed to both CO_2_ and human foot odor in comparison to all other treatments (*p* < 0.05; Figure 4A). No significant differences were observed among the other treatments.

#### Flight in Volume of Interest

When human host cues were present, mosquitoes spent a larger proportion of flight within the VOI (*p* < 0.05; Figure 4B). The highest proportion of flight within the VOI was elicited by the combination of CO_2_ and human foot odor, which was significantly different from all other treatments except human foot odor alone (Dunn Kruskal-Wallis test, *p* < 0.05). Stimulation with either human host cue on its own resulted in a significant increase of flight inside the VOI in comparison to the air control (*p* < 0.05), whereas there was no difference between air and clean sock control (*p* = 0.2).

#### Crosswind Flight

When analyzing the mean speed of mosquito crosswind flight for mosquitoes that were in contact to the VOI at least once (Figure 4C), a significant difference between the combination of both human host cues and air control (Dunn Kruskal-Wallis test, *p* = 0.01) and clean sock control (*p* = 0.01) was detected. Mosquitoes that were exposed to both CO_2_ and human foot odor flew on average 1.3 × faster in comparison to the air control. In addition, there was a tendency of increased crosswind flight frequency for both the human foot odor and the combination of human food odor with CO_2_ for larger distances to the source when pooling all mosquitoes (Supplementary Figure 3).

#### Landing and Landing Location

Of the 161 mosquitoes responding to the different treatments, 108 landed within the maximum recording time of 10 min. No significant difference was observed when comparing between treatments (*p* > 0.05; Figure 4D). However, the proportion of mosquitoes landing on target was significantly affected by the factors CO_2_ and human foot odor (Chi-square test, *p* < 0.001), in which 57% of the responsive mosquitoes landed on the target area on the upwind screen in response to CO_2_ and human foot odor, compared to 3% for the air and 10% for the clean sock control. These differences were significant among treatments (*p* < 0.01; Figures 4D,E).

### Case Study II—Carbon Dioxide

#### Response Rate

In response to the four CO_2_ treatments, 107 of 235 mosquitoes responded by entering the filmed volume. No significant effect of the concentration of CO_2_ was observed (GLM, Chi-square test; *p* = 0.2; Figure 5A).

#### Flight in Volume of Interest

No effect of the concentration of CO_2_ on the proportion of flight within the VOI was observed (Kruskal-Wallis test; *p* = 0.2; Figure 5B). The proportion of flight inside the VOI was generally low, ranging from 0.2% in response to 1,200 ppm to 2.9% for 2,400 ppm CO_2_.

#### Crosswind Flight

No significant difference in crosswind flight speed was observed when comparing between the treatments (Kruskal-Wallis test; *p* = 0.2; Figure 5C). There was no tendency of increased crosswind flight frequency between the treatments (Supplementary Figure 4).

#### Landing and Landing Location

Within the maximum recording time of 10 min, 77 of 101 mosquitoes landed. No significant difference was observed for the total proportion of mosquitoes landing (GLM, Chi-square test; *p* = 0.4), nor for the proportion of mosquitoes landing “on target” (*p* = 0.3; Figures 5D,E).

## Discussion

The two case studies presented here demonstrate the functionality of the versatile climate-controlled wind tunnel system as an experimental setup for analyzing insect flight. Moreover, we provide new findings and confirm previous observations on odor-mediated optomotor anemotaxis in *An. gambiae s. s*. Case study I recapitulates the characteristics of female *An. gambiae s. s*. host-seeking behavior in response to human host cues, as previously described in other contexts by 3D tracking studies (Spitzen et al., 2013; Hawkes and Gibson, 2016). In the present study, the combination of CO_2_ and human foot odor elicited a significant increase in mosquito responsiveness and host seeking, as reflected by a higher proportion of flight spent inside the volume where mosquitoes were more likely to encounter odor filaments. Moreover, mosquitoes tended to engage in more and faster crosswind flight in response to human odor cues. In addition, mosquitoes that responded to human host cues were also more prone to localize the odor source. This is consistent with previous studies on host seeking in both *An. gambiae* (Spitzen et al., 2013; Hawkes and Gibson, 2016) and other vector mosquito species (Cooperband and Cardé, 2006; Dekker and Cardé, 2011; Lacey and Cardé, 2011; van Breugel et al., 2015).

Similar to case study I, case study II took advantage of the isolated, thus human-odor-free, environment of the wind tunnel system and assessed the role of CO_2_ on its own as a host-seeking cue in *An. gambiae s. s*. There is currently a lack of consensus about the role of CO_2_ in eliciting activation, orientation and landing in *An. gambiae s. s*. females (de Jong and Knols, 1995; Healy and Copland, 1995; Takken et al., 1997; Spitzen et al., 2008; Lorenz et al., 2013; Webster et al., 2015). The concentrations of CO_2_ used in the present study are within the physiologically dynamic range of the CO_2_-sensitive neurons (Majeed et al., 2017), yet had no effect on responsiveness, crosswind flight, i.e., a measure for host seeking, or the accuracy of landing on the source. These findings are in accordance with previous observations in large-volume flight arenas, which demonstrate that *An. gambiae* do not rely on CO_2_ on its own to locate a human host (de Jong and Knols, 1995; Takken et al., 1997; Spitzen et al., 2008; see however Omondi et al., 2015; Majeed et al., 2017). Carbon dioxide is emitted by all hosts and is thus considered a general cue, signaling the presence of a host, but not necessarily the presence of a human (e.g., Mboera and Takken, 1997). For the highly anthropophilic *An. gambiae s. s*., CO_2_ is hypothesized to only contain information in the context of human odor (e.g., Takken and Verhulst, 2013). Such contexts include, e.g., the identification of presently inhabited human dwellings, by integrating CO_2_ with a persistent human odor-laden background, or, in the presence of multiple breathing hosts, the discrimination of host type, such as in dwellings shared by cattle and humans (Cardé and Willis, 2008; Webster et al., 2015). The latter has also been shown in mosquito species that demonstrate a wider breadth of host preference, in which the general host signal, emitted CO_2_, can be used as a reliable cue for host localization and discrimination (Dekker and Takken, 1998; Majeed et al., 2017).

The two case studies demonstrate the potential of the climate-controlled wind tunnel system to investigate the odor-mediated behavior of insects, including species that are sensitive to background odor contamination. The ability to finely adjust temperature and humidity, and to maintain these physical parameters at stable levels, provides the means to analyse the impact of future changes in climatic conditions on insect flight behavior. These parameters significantly affect population dynamics and vectorial capacity (Reiter, 2001; Paaijmans et al., 2010; Shapiro et al., 2017; Tang et al., 2018), but their effect on host seeking and other odor-mediated behaviors has until now not received any attention. The wind tunnel system provides additional means to analyse the effect of future anthropogenic changes in climate conditions on odor-mediated behaviors, as it requires no major modification to, for example, elevate background levels of greenhouse gasses, such as CO_2_ and ozone, as well as other atmospheric pollutants (Agrell et al., 2005; Majeed et al., 2014; Cook et al., 2020). Besides assessing the impact of future climatic changes on mosquito behavior, the future perspective for our laboratory is to evaluate the effectiveness of synthetic blends as attractants in mosquito control and monitoring devices. Moreover, we envision that fine-scale analysis of behavior will provide valuable information on how the peripheral and central olfactory systems detect and integrate olfactory information.

## Data Availability Statement

The raw data supporting the conclusions of this article will be made available by the authors, without undue reservation.

## Author Contributions

AH, SH, and RI conceived and designed the experiments. JL designed and constructed the wind tunnel system. AH established the experimental system, performed the experiments, and analyzed the results. AH drafted the manuscript and AH, SH, and RI critically revised the manuscript. All authors approved the final version of the manuscript.

## Conflict of Interest

JL was employed by company Jörgen Lantz Engineering Consulting Firm. The remaining authors declare that the research was conducted in the absence of any commercial or financial relationships that could be construed as a potential conflict of interest.
