# Supplementary material for: Mosquito Host Seeking in 3D Using a Versatile Climate-Controlled Wind Tunnel System
Source: Front Behav Neurosci. 2021 Mar 11;15:643693. doi: 10.3389/fnbeh.2021.643693 (PMC7991727; doi:10.3389/fnbeh.2021.643693)
Supplement: Supplementary file 1 [file Data_Sheet_1.PDF]

Supplementary Material

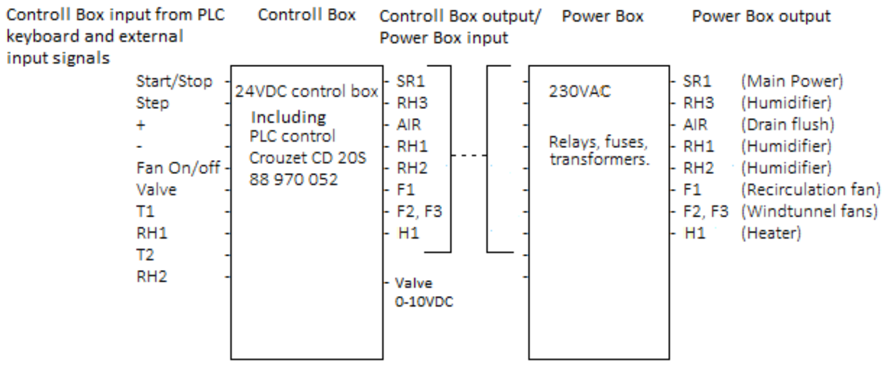

Supplementary Figure S1. Circuit diagram of the control panel.

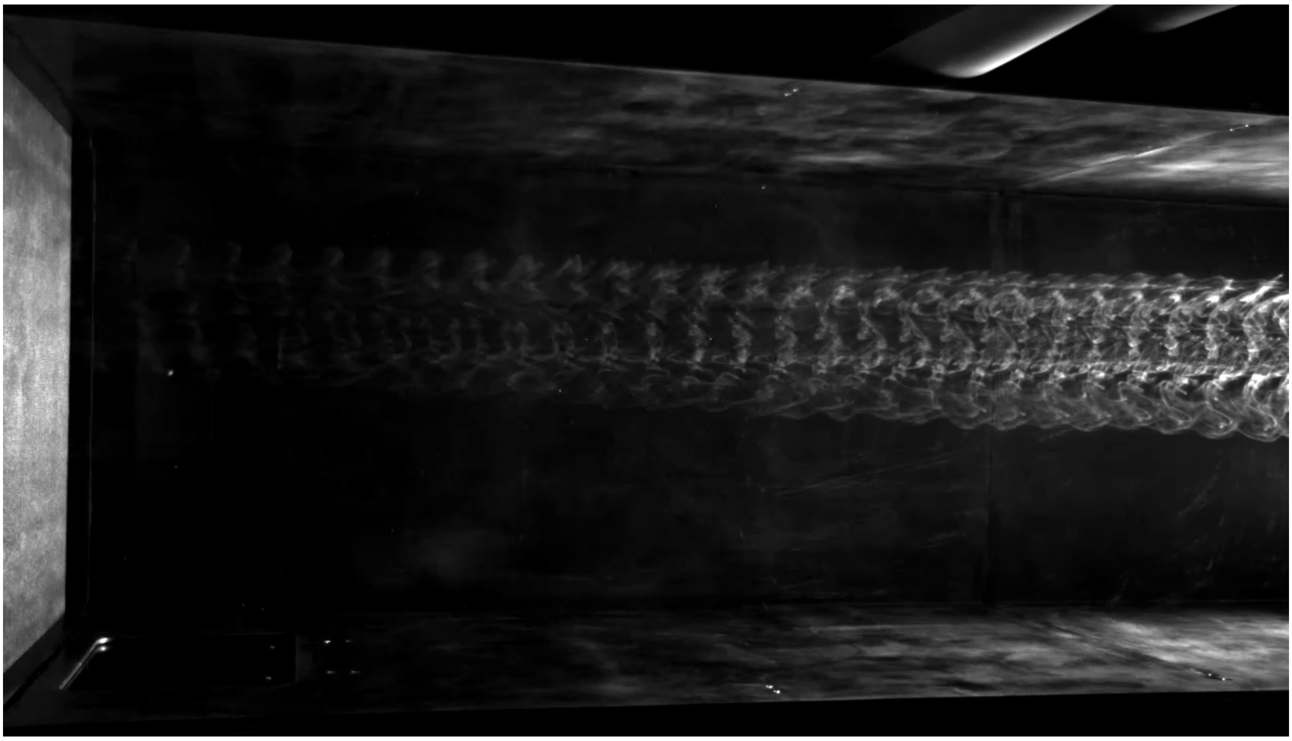

Supplementary Figure S2. Shape and dimensions of the turbulent plume that is generated by the glass hoop, visualised by smoke paper. Note that the illumination used for this image was placed at the end of the flight arena, thus the plume is not well illuminated close to the upwind screen (left). The glass hoop was not centred for this picture.

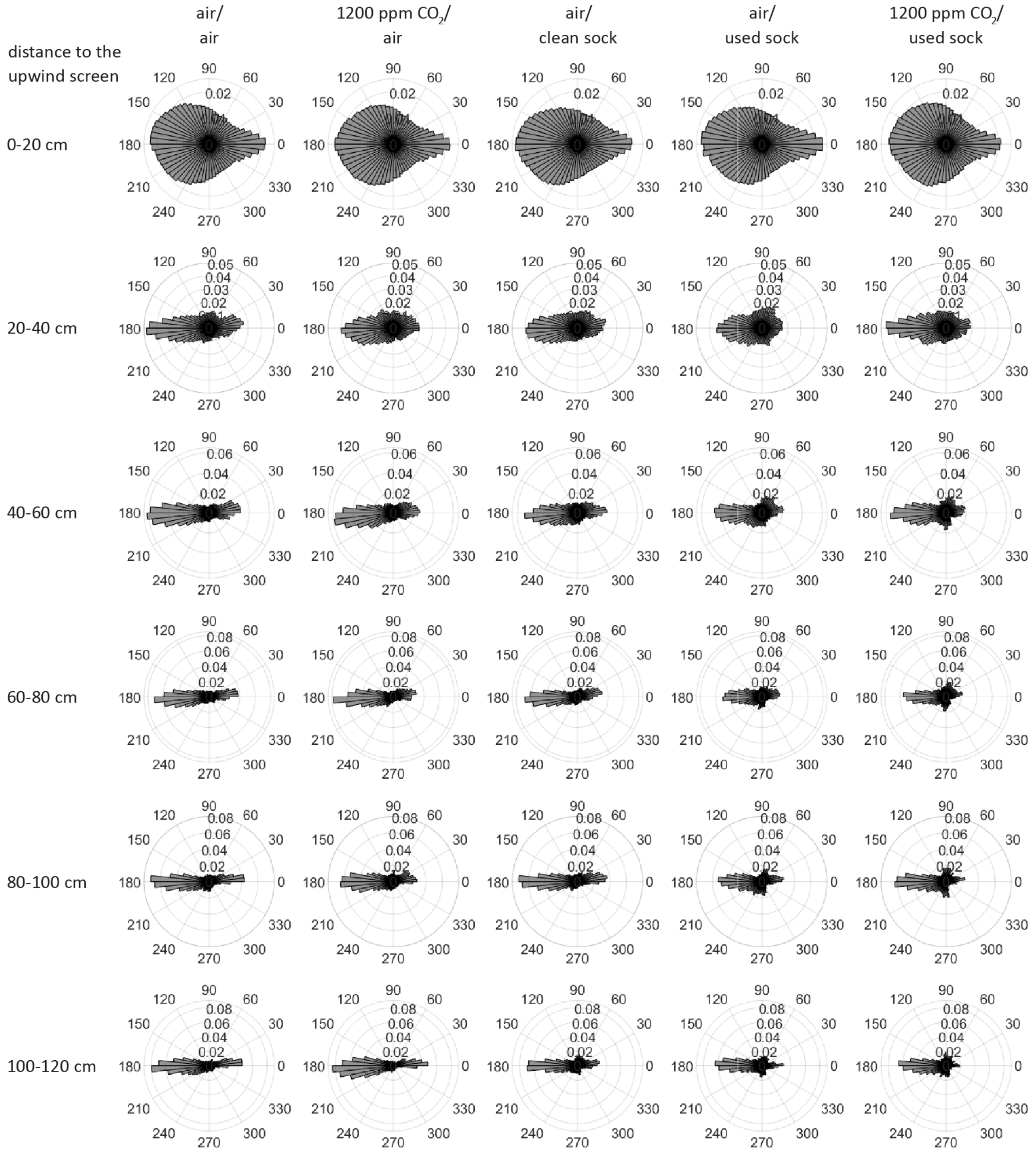

Supplementary Figure S3. Polar histograms of mosquito flight in response to human host cues. Bars show the relative number of heading angle groups (bin width 6°) in the vertical plane as a measure for the predominant flight direction. 180° corresponds to straight upwind flight. Each treatment is divided into sections, corresponding to the distance of the mosquito to the upwind screen. The width of each section is 20 cm.

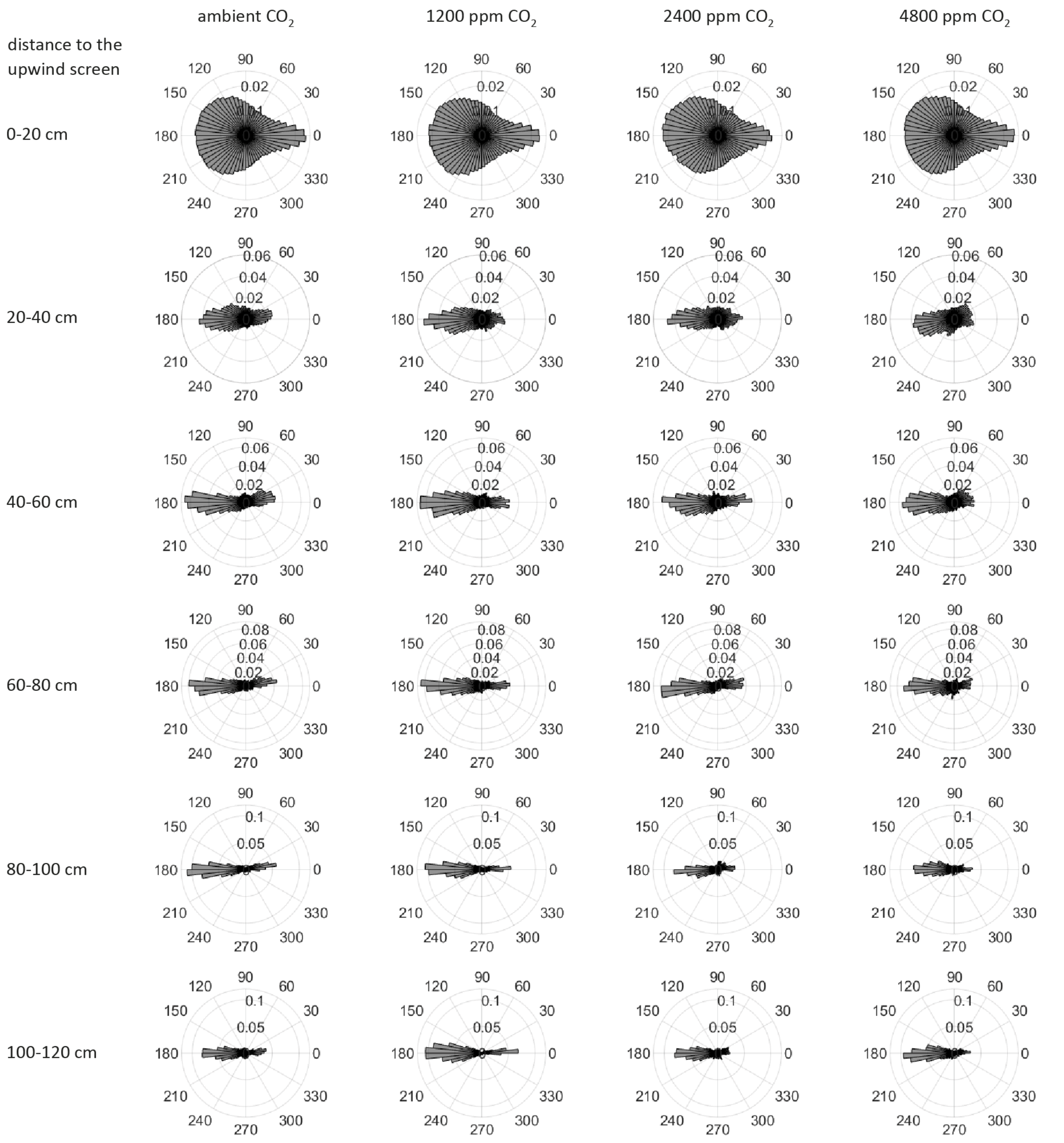

Supplementary Figure S4. Polar histograms of mosquito flight in response to different concentrations of carbon dioxide (CO_2). Bars show the relative number of heading angle groups (bin width 6°) in the vertical plane as a measure for the predominant flight direction. 180° corresponds to straight upwind flight. Each treatment is divided into sections, corresponding to the distance of the mosquito to the upwind screen. The width of each section is 20 cm.
